# Demonstrating an Academic Core Facility for Automated Medical Image Processing and Analysis: Workflow Design and Practical Applications

**DOI:** 10.3390/diagnostics15070803

**Published:** 2025-03-21

**Authors:** Yogesh Kumar, Rex A. Cardan, Ho-hsin Chang, Katherine A. Heinzman, Kadir Gultekin, Amy Goss, Andrew McDonald, Donna Murdaugh, Jonathan McConathy, Steven Rothenberg, Andrew D. Smith, John Fiveash, Carlos E. Cardenas

**Affiliations:** 1 Department of Radiation Oncology, University of Alabama at Birmingham, Birmingham, AL 35233, USA; rcardan@uabmc.edu (R.A.C.); changh@uab.edu (H.-h.C.); kheinzman@uabmc.edu (K.A.H.); ammcdonald@uabmc.edu (A.M.); jfiveash@uab.edu (J.F.); 2 Department of Biomedical Engineering, University of Alabama at Birmingham, Birmingham, AL 35294, USA; gultekin@uab.edu; 3 Institute for Cancer Outcomes and Survivorship, University of Alabama at Birmingham, Birmingham, AL 35233, USA; dlmurdaugh@uab.edu; 4 Department of Nutrition Sciences, University of Alabama at Birmingham, Birmingham, AL 35233, USA; amymiski@uab.edu; 5 Department of Pediatrics, University of Alabama at Birmingham, Birmingham, AL 35233, USA; 6 Department of Radiology, University of Alabama at Birmingham, Birmingham, AL 35294, USA; jmcconathy@uabmc.edu (J.M.); srothenb@uab.edu (S.R.); andrew.smith@stjude.org (A.D.S.); 7 Department of Radiology, St Jude Children’s Research Hospital, Memphis, TN 38105, USA

**Keywords:** advanced medical imaging workflows, imaging core facility

## Abstract

**Background/Objectives:** Medical research institutions are increasingly leveraging artificial intelligence (AI) to enhance the processing and analysis of medical imaging data. However, scaling AI-driven medical image analysis often requires specialized expertise and infrastructure that individual labs may lack. A centralized solution is to establish a core facility—a shared institutional resource—dedicated to Automated Medical Image Processing and Analysis (AMIPA). **Methods:** This technical note offers a practical roadmap for institutions to create an AI-based core facility for AMIPA, drawing on our experience in building such a resource. **Results:** We outline the key components for replicating a successful AMIPA core facility, including high-performance computing resources, robust AI software pipelines, data management strategies, and dedicated support personnel. Emphasis is placed on workflow automation and reproducibility, ensuring researchers can efficiently and consistently process large imaging datasets. **Conclusions:** By following this roadmap, institutions can accelerate AI adoption in imaging workflows and foster a shared resource that enhances the quality and productivity of medical imaging research.

## 1. Introduction

The integration of AI into medical image processing and analysis is expanding significantly, enabling faster and more quantitative insights from imaging data [1,2]. However, many research institutions face challenges in scaling AI for imaging due to substantial demands for computational resources and specialized expertise [3]. Individual research groups often face the continuous expansion of imaging datasets but lack robust automated analysis solutions, underscoring a gap that must be addressed [4].

A promising approach to bridge this gap is the creation of a dedicated core facility for AI-driven image analysis. For research purposes, a core facility is a centralized, shared resource offering access to advanced instruments, technologies, and expert services throughout an institution [5,6,7,8]. These facilities have become essential in modern science, enabling investigators to access sophisticated capabilities that would be challenging for individual labs to develop independently. Core facilities now play a critical role in biomedical research by providing scientists access to cutting-edge technology and expertise [9].

In the field of medical imaging, an AI-based core facility can significantly accelerate research workflows by centralizing high-performance computing and AI algorithms in a single hub [10]. For example, the University of Utah’s Biomedical Imaging, Data Science, and AI Core (BIDAC) functions as a health sciences core facility, offering advanced AI-driven image analysis services to academic research groups [11]. BIDAC’s model illustrates how a dedicated imaging AI core facility can complement existing research infrastructure and support a broad community of users. Inspired by this approach, many institutions are now interested in establishing their own AI-powered imaging core facilities.

However, building an AI-centric imaging core facility from the ground up presents significant challenges. Institutions must identify the appropriate hardware and software infrastructure, assemble a team with the necessary expertise, integrate with data workflows (often including clinical systems), and establish sustainable operational practices [10,12,13]. There is a clear need for a comprehensive roadmap that outlines these steps in detail to guide organizations through the process.

**Figure 1:**
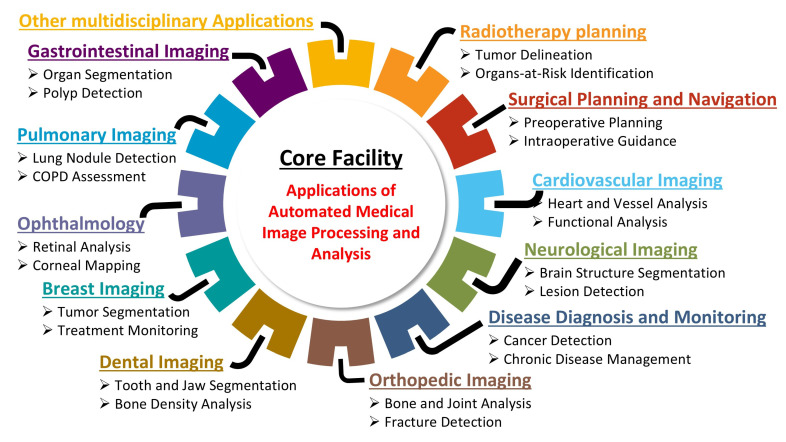
Multidisciplinary applications of *AMIPA* across healthcare specialties.

This technical note provides a step-by-step roadmap for establishing an AI-based core facility for AMIPA that can support a wide range of investigators and subspecialties (Figure 1). It is organized into three main sections that guide the reader through the process. Section 2 (Materials and Methods) outlines the design and implementation of the core facility, detailing the required infrastructure, automated imaging workflows, personnel training, and key considerations such as funding and regulatory compliance to ensure efficient operation. Section 3 (Case Studies) showcases the facility in action through a range of case studies across multiple medical domains, illustrating how its AI-driven pipelines support various research applications. For example, projects ranging from analyzing body fat distribution in obesity research to monitoring brain volume changes in cancer survivors highlight how the facility’s automated image analysis enhances both processing efficiency and result accuracy. Finally, Section 4 (Discussion and Conclusions) reflects on the broader impact and lessons learned from our experience, discussing the facility’s contributions to research efficiency, the challenges encountered during its development, and future directions for integrating AMIPA into clinical and research settings.

### 1.1. Statement of Significance

#### 1.1.1. Problem or Issue

There is limited guidance available for academic and research institutions on effectively implementing core facilities for AMIPA, leading to challenges related to expertise and infrastructure.

#### 1.1.2. What Is Already Known

Various AI-based core facilities in medical imaging have shown potential in improving diagnostic workflows, supporting multimodal image analysis/synthesis, and enhancing image accuracy. Despite these advances, challenges remain in integrating AI with existing clinical infrastructure and ensuring sustainable facility management for long-term operational effectiveness.

#### 1.1.3. What This Technical Note Adds

This technical note provides a roadmap for an AI-based core facility model designed for AMIPA, offering a structured workflow that includes the automatic detection of image format and its preprocessing to ensure compatibility, automatic selection of pretrained AI model, and an automated pipeline that allows collaborators to upload imaging data and automatically runs AI-based medical image analysis via protected data repository, addressing common operational challenges. Our approach demonstrates practical applications of AI across imaging tasks and outlines funding strategies, collaborative practices, and technological integrations that make our core facility sustainable and adaptable. This framework serves as a guide for institutions looking to implement or optimize similar facilities in academic and clinical settings.

## 2. Materials and Methods

### 2.1. Establishing the Core Facility

The development of our core facility was initially supported by funding from our institution, providing the financial resources to lay the groundwork for its establishment. We began by acquiring specialized equipment for high-throughput medical image processing and analysis, ensuring that our facility could deliver cutting-edge services. At the same time, we assembled a skilled team of personnel with expertise in AI, medical imaging, and related fields, implementing comprehensive training programs to equip them with the necessary skills to operate the facility effectively. This foundational phase was essential in setting up a robust infrastructure capable of supporting diverse research and clinical needs.

### 2.2. Equipment

To meet the computational demands of our core facility, we invested in servers and workstations specifically designed for running production services and staging pre-deployment processes (see Table 1). These servers provide ample central processing unit (CPU) and Graphics Processing Unit (GPU) parallelization to handle a wide variety of complex medical image processing and analysis tasks, ensuring both high throughput and reliability.

AMIPA’s software stack runs on Ubuntu 22.04.2 LTS (64-bit) for stability and compatibility with deep-learning frameworks, ensuring efficient performance for our automated image analysis tasks (See Supplementary Materials File S1). We utilized batch processing, Digital Imaging and Communications in Medicine (DICOM) compression, and multithreaded data pipelines to efficiently process large volumes of medical images. All our projects are containerized using Docker, allowing a seamless transition from development to production servers. We collaborated closely with our informational technology (IT) team to host these servers within our institutional data centers and ensure that they adhered to institutional guidelines and security standards. Our production and staging servers are equipped with identical hardware to ensure consistent system integration and provide a reliable backup in case of a production system malfunction. This approach minimizes compatibility issues and allows for smooth transitions between environments, enhancing both operational efficiency and system resilience by minimizing any potential service downtime. Our collaboration with the IT team extends beyond the initial server installation and configuration; they are also responsible for managing the security, updates, and overall servicing of all hardware equipment to minimize downtime.

Additionally, we outfitted the facility with dedicated workstations for development, providing our team with the hardware and software tools needed to refine and enhance AI algorithms and workflows. Our facility is also integrated with the institutional high-performance computing cluster, Cheaha, which offers the additional computational power required for handling large-scale data processing.

### 2.3. Personnel

The success of a core facility is heavily dependent on the expertise and dedication of its personnel. We assembled a multidisciplinary team that includes specialists in AI, medical imaging, network security, and regulatory compliance. The core facility director oversees strategic planning and daily management. AI specialists develop and optimize machine learning models tailored for AMIPA, ensuring accuracy and efficiency. Medical imaging experts provide clinical insights to validate AI-driven analyses and verify alignment with radiological standards and clinical relevance. Software engineers manage data pipelines, integration, and deployment of AI tools. IT and network security specialists oversee data protection, Health Insurance Portability and Accountability Act (HIPAA) compliance, and system integrity to safeguard patient information and research data. Regulatory compliance experts affirm adherence to institutional, federal, and international guidelines, assisting with ethical approvals and the necessary documentation for AI-driven medical research. Each team member brings a wealth of experience and knowledge, confirming that we can effectively address the diverse needs of the core facility’s users. Continuous professional development is a priority; we provide opportunities for our personnel to attend workshops, conferences, and training sessions to stay up to date with the latest advancements in their respective fields. This commitment to fostering a skilled and knowledgeable team is essential for delivering high-quality services and maintaining the operational excellence of our core facility.

### 2.4. Training and Education

To ensure the effective operation and utilization of the core facility, we implemented comprehensive training programs tailored to the diverse needs of our staff and users. These programs address several key areas: the technical operation of equipment, the application of AI algorithms in medical image processing and analysis, and the adherence to regulatory standards. The multimodal image acquisition equipment includes X-ray, Computed Tomography (CT), magnetic resonance imaging (MRI), Positron Emission Tomography (PET)-CT, and others. Additionally, open-source software tools, such as 3D Slicer, Dicompyler, and ITK, play an important role in analyzing and visualizing both preprocessed and postprocessed data during design and development phases [14,15,16]. Moreover, training utilizing AI-driven medical imaging algorithms (e.g., nnUNet, Synthseg, etc.) across various resolution settings (full resolution, low resolution, 2D, etc.) for specific regions of interest (ROIs), organs, and body tissues helps our personnel address specific clinical needs, as outlined below.

⮚ Enhance technical proficiency (training, fine-tuning, and deploying AI model);
⮚ Improve troubleshooting and model optimization skills (identify and resolve issues, adjusting model parameters);
⮚ Streamlined workflow for automated pipelines;
⮚ Learn better data management and preprocessing skills (data augmentation, normalization, and model validation);
⮚ Efficient model deployment and clinical translation (evaluate AI outputs and validate results).

Our training program includes hands-on workshops, online tutorials, and one-on-one meetings to ensure end users gain a comprehensive understanding of the developed algorithms, with a particular focus on each tool’s intended use and limitations. Additionally, specialized training sessions are dedicated to critical aspects of managing service requests, including regulatory compliance, project design, project development, system integration, and deployment. By offering ongoing education and support in these areas, we aim to equip our personnel with the skills and knowledge required to maintain high service standards, foster innovation, and ensure the successful implementation and management of our core facility’s capabilities.

#### 2.4.1. Managing Service Requests

**Figure 2:**
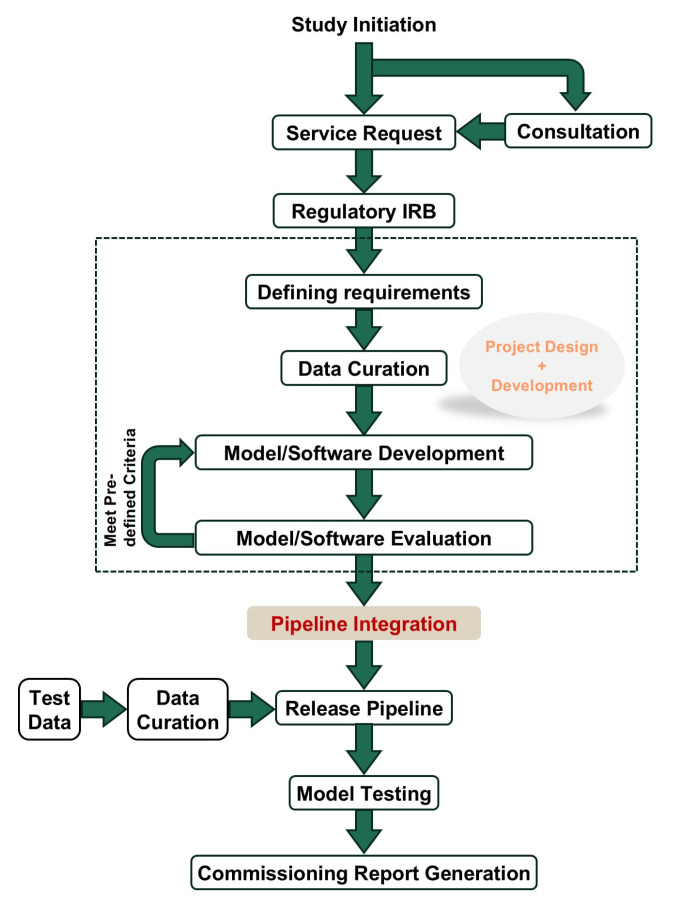
Detailed workflow of design, development, and integration in the AMIPA core facility.

Effective management of service requests is essential for the smooth operation of a core facility and user satisfaction. Our approach integrates several key components: regulatory compliance, project design, development, integration, and deployment (Figure 2). We ensure that all requests comply with regulatory standards, prioritizing data security and patient confidentiality. Using the IEEE Computer Society’s SWEBOK Guide v4 (2024) as a framework, we define project requirements in collaboration with users to confirm alignment with research objectives [17]. This approach not only customizes solutions to meet specific needs but also establishes clear timelines and sets expectations for project completion. By doing so, we create a structured process that enhances transparency, ensures projects are delivered efficiently, and fosters a more collaborative and productive research environment in AMIPA.

##### Regulatory

In managing service requests, adherence to regulatory standards is paramount to ensure compliance and safeguard data integrity. Our core facility strictly follows all applicable regulations related to medical imaging and data management, including privacy laws, ethical guidelines, and institutional policies. We implement robust protocols for data handling and storage to maintain confidentiality and security, and we regularly review these protocols to stay current with evolving regulations. Our team collaborates with the institutional review board (IRB) to ensure that all practices align with approved protocols’ guidelines. Two approaches to regulatory oversight in core facilities include relying on individual principal investigators’ IRBs for project-specific reviews, verifying adherence to protocol requirements, or establishing a single, broad IRB protocol dedicated to the facility. This broad IRB can streamline processes for routine analyses, providing a standardized framework that meets regulatory standards while enhancing efficiency across multiple projects. By prioritizing regulatory adherence, we not only protect sensitive information but also build trust with those using our services, fostering a reliable and secure environment for AMIPA-based research.

##### Project Design

The project design phase is critical for establishing a streamlined workflow for AMIPA. It begins with an initial consultation to understand the specific needs of the research study, followed by a formal service request that outlines the objectives, desired outcomes, and required resources. Using the SWEBOK Guide, project requirements are carefully defined in collaboration with the user. This includes specifying key deliverables, timelines, resources, and technical specifications, ensuring that the project aligns with both research objectives and operational feasibility [17]. The request is then thoroughly reviewed to affirm compliance with the IRB-approved protocol. Once approved, the project enters the quality control phase, where input data are meticulously reviewed for accuracy and completeness (i.e., data curation). The analysis pipeline is then designed, involving the selection of in-house developed AI-based algorithms and/or open-source solutions. Subsequently, the output format is determined based on researchers’ needs. After the pipeline is established, the results from automated analyses undergo quality assurance to validate system’s performance and reliability. This process generates software commissioning reports and documentation, ensuring the core facility operates efficiently, meets high research standards, and delivers reliable, high-quality data for analysis and interpretation. This detailed workflow enables the core facility to effectively manage service requests effectively, seamlessly integrate new projects, and support the ongoing development and deployment of AI-based advanced medical image processing and analysis techniques.

##### Project Development

The project development phase builds on the foundation established during the project design phase, focusing on implementing and refining the analysis pipeline. It begins with setting up the computational infrastructure, including configuring servers, installing necessary software, and integrating AI-based algorithms. During this phase, collaboration with data scientists, radiologists, and IT specialists ensures that the pipeline meets the specific needs of the research study. Key tasks include data preprocessing to clean raw medical images and the customization of algorithms to optimize their performance. Continuous testing and iterative improvements further enhance pipeline accuracy and efficiency.

Additionally, we adhere to the recommendations of the American Association of Physicists in Medicine (AAPM) and other relevant societies to verify that the required accuracy and standards are met before approving the pipeline [18,19,20,21,22]. This stage also involves the development of user interfaces and tools to facilitate easy interaction with the pipeline, run analyses, and retrieve results seamlessly. Throughout the project development phase, robust documentation practices are maintained to record modifications, parameter settings, and troubleshooting steps to ensure transparency and reproducibility. The culmination of this phase is the creation of a fully functional and validated analysis pipeline ready for deployment.

##### Integration

The integration phase is critical for ensuring the newly developed AMIPA pipeline seamlessly integrates into the core facility’s existing infrastructure and workflows. This involves interfacing the pipeline with various data management systems to facilitate smooth data transfer and ensure interoperability between different software and hardware components. To enhance integration, all pipelines are developed using Docker (v28.0.1) containers, which provide a consistent and isolated environment for the software to confirm reliable operation across different systems and minimize conflicts with existing applications and dependencies. Comprehensive testing validates the integration process, confirming that the pipeline can process data from diverse sources and produce consistent, accurate results. Collaboration with IT specialists and end-users addresses compatibility issues and optimizes system performance. Effective integration enhances the core facility’s capabilities, equipping researchers with reliable and efficient AI-based tools for advanced medical image processing and analysis.

##### Deployment

The deployment phase marks the transition of the analysis pipeline from development to operational use within the core facility. This phase involves installing the validated pipeline onto the production environment, ensuring all components are properly configured and fully functional. Support resources, including user manuals and troubleshooting guides, are provided to assist with any initial challenges. During deployment, continuous monitoring is implemented to promptly identify and resolve any technical issues, minimizing disruption to ongoing research activities. Feedback from early users is actively gathered to make any necessary adjustments and improvements. Successful deployment confirms that the pipeline operates efficiently, delivering high-quality, reliable data for medical image processing and analysis and significantly enhancing the research capabilities of the AMIPA core facility.

#### 2.4.2. Promoting the Core Facility

Promoting the core facility is essential for attracting researchers and collaborators who can benefit from its AMIPA capabilities. A comprehensive marketing strategy can be employed to raise awareness and highlight the facility’s offerings. Engaging in social media campaigns and leveraging professional networks on platforms like LinkedIn helps reach a wider audience. Additionally, presenting at conferences, seminars, and workshops provides opportunities to showcase the facility’s capabilities and foster new partnerships. Collaborations with academic institutions and industry leaders can further expand the facility’s reach and reputation. Regular newsletters and email campaigns can help keep the research community informed about new developments, services, and upcoming events. Effective advertising ensures that the core facility is recognized as a leading resource in the field, attracting a diverse range of projects and fostering a dynamic research ecosystem.

In our case, this marketing strategy has resulted in positive word-of-mouth recommendations from colleagues who have shared their experiences with our facility’s services. This informal promotion has helped us strengthen our reputation and attract more users. Additionally, maintaining a dedicated website for the core facility has proven invaluable, as it centralizes information and resources, making it easier for potential users to access and learn about our services. This online presence has further supported our efforts to engage with the research community and reach new users.

#### 2.4.3. Maintenance and Long-Term Outlook

Ensuring the sustainability and longevity of our core facility requires a robust maintenance strategy and a forward-thinking outlook. Regular maintenance schedules are established for all equipment and systems to minimize downtime and maintain optimal performance. This includes routine inspections, software updates, and hardware servicing, all conducted in collaboration with our institutional IT and technical support teams. To stay at the forefront of AMIPA, we continuously evaluate and integrate emerging technologies and methodologies.

## 3. Case Studies

**Figure 3:**
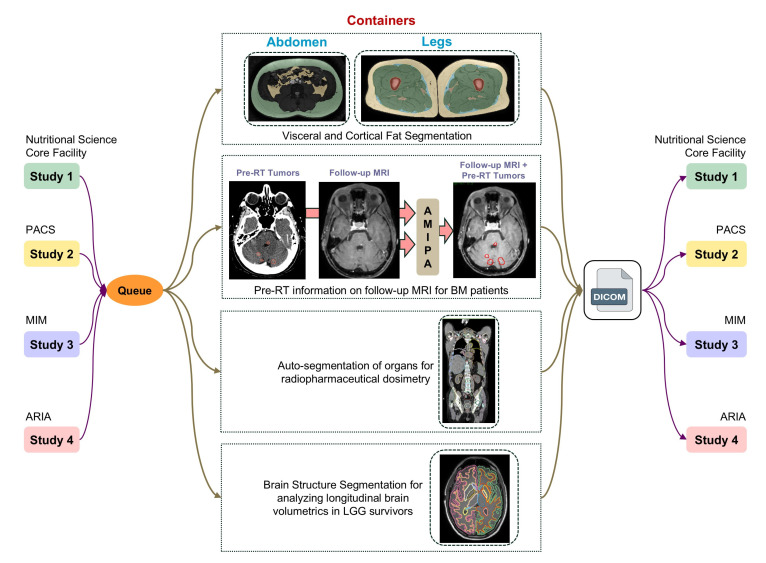
Leveraging published pipelines: researcher access to AMIPA facility-based containers. This figure illustrates the workflow allowing researchers to connect with the AMIPA facility’s deployed containers for customized case-specific analysis using standardized pipelines. Each pipeline is exclusively available to individual researchers, ensuring data confidentiality and appropriate reporting throughout the research process. This figure highlights our established integration across multiple clinical and research systems (Picture Archiving and Communication System (PACS), ARIA Oncology Information System (Varian Medical Systems, Palo Alto, CA, USA), MIM (MIM Software, Cleveland, OH, USA), etc.) within our institution, enabling streamlined workflows and data accessibility.

**Figure 4:**
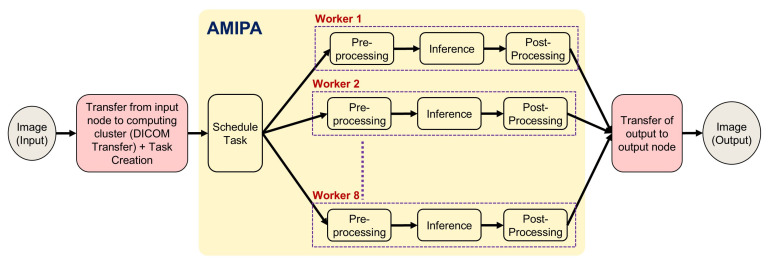
The AMIPA queuing process shows how inferences are drawn from an input node to an output node via a DICOM daemon. Although each study has a unique input node, all studies share the same queue, and tasks are scheduled and executed based on worker availability.

In this section, we present recent case studies that demonstrate the practical applications and impact of our institution’s AMIPA core facility on various research projects. These examples highlight the versatility and effectiveness of our services in addressing complex research challenges across multiple disciplines. Specifically, we explore studies focusing on abdominal and leg fat analysis, longitudinal brain volumetrics in glioma survivors, volumetric brain assessment in long-term head and neck cancer (HNC) survivors, and auto-segmentation of organs for dosimetry. The workflow of how researchers collaborate with our facility—sending raw images for analysis and receiving processed AMIPA-based results—is illustrated in Figure 3. The queuing process follows the same procedure for all studies (Figure 4). First, (1) input images (DICOM) are transferred from the medical imaging system to a secured shared repository via a DICOM daemon. Each study is associated with a unique node. Next, (2) the queue manager creates or schedules a task through a task listener, which (3) triggers the defined pipeline steps, typically including pre-processing (e.g., DICOM-to-NIFTI conversion, normalization, selecting window width and level for CT images, and resampling to isotropic voxels), inference (e.g., AI models for leg/abdomen fat segmentation or brain structure segmentation), and post-processing (e.g., morphological operations, connected component analysis, feature extraction, statistical metrics like Dice Similarity Coefficient (DSC), Hausdorff distance (HD), Mean Surface Distance (MSD), and report generation). (4) Relevant metrics and results are extracted, and (5) the data are then converted back to DICOM format. Finally, (6) the AI-driven output data are transferred to the designated output node.

### 3.1. Automated Analysis of Abdominal and Leg Fat Composition for Obesity Studies

Obesity, characterized by an excess accumulation of body fat, is linked to the development of chronic metabolic diseases such as type 2 diabetes (T2D), cardiovascular disease, and metabolic dysfunction-associated steatotic liver disease (MASLD) in both adults and children [23,24]. However, obesity is a heterogeneous condition, and the location or distribution of adipose tissue may be a more relevant factor in assessing the risk of metabolic disease [25]. For instance, the deposition of adipose tissue in the visceral cavity and skeletal muscle is independently associated with insulin resistance and poor cardiometabolic outcomes, while a greater fat distribution in the periphery areas is associated with lower disease risk [26]. A collaboration with faculty in the Department of Nutrition and the National Institutes of Health (NIH)-funded Nutrition Obesity Research Center Sciences focused on analyzing changes in visceral and ectopic fat in response to diet interventions. Our facility was used for the continuous monitoring of fat depots in the abdomen (visceral adipose tissue and abdominal subcutaneous adipose tissue) and legs (thigh subcutaneous adipose tissue, thigh perimuscular adipose tissue, and intermuscular adipose tissue) using MRI. In their previous workflow, abdominal and leg fat were manually annotated on individual imaging studies for each patient. Our team developed a pipeline that automates the segmentations of abdominal and leg fat using the widely validated nnUNet framework. nnUNet is a powerful deep-learning tool known for its high accuracy in multimodality medical image segmentation [27]. For this task, we leveraged previously annotated datasets and iteratively trained a 2D model for each abdomen and leg fat segmentation. Automating this process significantly reduces the amount of manual effort required per patient, offering considerable advantages, particularly for longitudinal studies.

### 3.2. Improving Post-RT Follow-Up for Brain Metastases Patients: An AI-Based Solution

Recently, collaborators from the Department of Radiation Oncology at our institution conducted a clinical study aimed at improving the post-RT assessment workflow for patients with brain metastases (BMs) [28]. These patients frequently undergo multiple courses of RT, making post-treatment evaluations time-consuming. This process typically requires clinical teams to review follow-up imaging alongside the corresponding RT plan history. The goal of this study was to develop a fully automated solution that integrates previously delivered RT plan information with follow-up MRI imaging, enhancing both the efficiency and accuracy of this critical evaluation process and allowing for better differentiation between radiation-induced injury, local recurrence, and new BMs.

Our core facility played a pivotal role in this research by performing automatic segmentation and automatic registration between planning CT and follow-up MR images. This automated workflow was tested on 20 patients who had previously undergone multiple courses of stereotactic radiosurgery. When comparing the accuracy of manual versus automated rigid registrations, the results showed strong agreement across all evaluated contours. Overall, the automated registrations exhibited high accuracy, with DSC values demonstrating strong agreement for most organs and tissues. Additionally, MSD measurements showed a minimal discrepancy in automatically registered brain structures. These results highlight the effectiveness of the core facility’s automated workflow, showcasing its ability to achieve precise and reliable registrations with broader potential for clinical research applications beyond volumetric analysis.

### 3.3. Auto-Segmentation of Organs for Radiopharmaceutical Dosimetry

In a recent study conducted by collaborators in the Division of Molecular Imaging and Therapeutics at UAB’s Department of Radiology, researchers explored the potential of [^89^Zr]Zr-oxine labeled autologous leukocytes for PET imaging to overcome the limitations of traditional tools in tracking brain-infiltrating leukocytes in living humans. While SPECT radiopharmaceuticals like [^111^In]In-oxine and [^99m^Tc]Tc-hexamethylpropylenamine-oxime (^99m^Tc-HMPAO) have proven useful in this application, PET imaging offers higher sensitivity, spatial resolution, and temporal resolution, prompting the development of PET-based techniques for autologous leukocyte labeling [29,30,31,32,33,34,35,36]. For longer-lived radiopharmaceuticals like [^89^Zr]Zr-oxine leukocytes, human dosimetry estimates are required prior to routine use in human research studies.

An IRB-approved study at our institution set out to determine the dosimetry of [^89^Zr]Zr-oxine leukocytes. In this study, four adult female subjects were administered [^89^Zr]Zr-oxine labeled autologous leukocytes, followed by whole-body PET/CT imaging at multiple intervals (0, 4, 24, 48, and 72 h post-administration). Our AMIPA core facility played an important role by providing automated workflows for segmenting over 150 organs and tissues on non-contrast whole-body CT scans, which served as the basis for determining patient-specific internal organ dosimetry for this study.

### 3.4. Brain Structure in Glioma Survivors

Gliomas are brain tumors that originate from glial cells, which are responsible for nourishing neurons in the brain [37]. Radiotherapy (RT) is commonly used as a treatment option for low-grade glioma (LGG) patients, but it can also result in cognitive effects in LGG survivors [38]. Several studies have highlighted the volumetric changes in brain anatomy following RT treatment of LGG [38]. Our collaborators at the Institute for Cancer Outcomes and Survivorship within our institution hypothesized that LGG survivors may experience significant brain volume loss after RT treatment [38].

To characterize volume changes, clinical records from 105 LGG patients treated between October 2004 and March 2021 were collected. These patients received either conformal RT or intensity-modulated RT. To assess brain volume changes, our core facility evaluated the use of SynthSeg (v2.0) for brain sub-structure auto-segmentation [39]. Due to the absence of ground-truth segmentation data in our institutional dataset, we relied on a previously defined 5-point Likert scale to qualitatively review the resulting contours, thereby making the analysis more readily applicable. As noted by McDonald et al., we found that SynthSeg-generated ROIs generally met clinical acceptability in multi-modality 3D MR images. After validating the accuracy of this open-sourced tool, we processed each patient’s follow-up MRI scans (median = 12, range: 2–40) through the SynthSeg pipeline and converted its output to RT DICOM format for editing in our institution’s clinical treatment planning system. In total, over 1000 follow-up scans were processed through our pipeline without user interaction, as the pipeline was directly connected to our clinical PACS server and run through DICOMAnon (Red Ion LLC), a commercially available DICOM anonymization solution, prior to entering our pipeline. Had this process been performed manually, contouring brain substructures would have taken approximately 30 min per MRI scan, making such extensive analysis nearly unfeasible.

Our collaborators found that after two years of RT treatment, significant changes in brain volumes were observed: a reduction of −4.9% for cortical white matter (*p* = 0.038) and −3.1% for the hippocampus (*p* = 0.041). The effects of RT treatment were further quantified using the slope (β) from a hierarchical linear model (HLM), which revealed a progressive loss of brain volume over time. This comprehensive analysis was made possible through the advanced capabilities and efficiency of our core facility’s services.

A similar study by Murdaugh et al. on HNC survivors, conducted with the support of our AMIPA core facility, employed voxel-based morphometry (VBM) and statistical parametric mapping (SPM12) to monitor volumetric changes in brain structures [40]. This study provided further evidence of significant brain volume changes in specific regions, particularly highlighting the radiation dose’s inverse relationship with gray matter (GM) volumes in regions such as the left ventral diencephalon.

## 4. Discussion and Conclusions

In this technical note, we share our experience by discussing the operational workflow and impact of our institution’s AMIPA core facility, including use cases where the facility has provided critical research support across multiple medical domains. Our goal is to provide a roadmap for the medical imaging research community to establish and maintain similar core facilities. We also showcase the facility’s utility through case studies, demonstrating its ability to enhance both research outcomes and clinical practices. Despite being in the first 18 months since establishment, our core facility has already built a strong track record of delivering valuable research services, resulting in increased funding opportunities for our facility through recently submitted grant proposals and fostering academic–industry partnerships.

Establishing comprehensive processes within our core facility has been instrumental in significantly improving operational efficiency, increasing throughput, and enhancing the quality of research data. By strategically investing in high-performance equipment tailored for high-throughput medical image processing and analysis and by collaborating closely with our IT team, we have built a robust and secure infrastructure that minimizes downtime and ensures seamless integration of new technologies. Our multidisciplinary team effectively manages service requests by adhering to regulatory standards and utilizing structured frameworks like the IEEE SWEBOK Guide for project design, development, integration, and deployment. The use of standardized tools such as Docker containers has streamlined the integration of AI-based pipelines, further improving system reliability and performance. Additionally, proactive promotion and maintenance strategies, including collaborations and continuous professional development, have expanded our facility’s reach and ensured its long-term sustainability. Together, these established processes not only accelerate project timelines and increase data throughput but also enhance data integrity and reproducibility, making a substantial contribution to the advancement of research in AMIPA.

The utility of our core facility was demonstrated through several case studies, including the automated analysis of abdominal and leg fat composition in obesity and nutrition studies, auto-segmentation of organs for dosimetry, and the application of advanced AI-based solutions to improve post-RT follow-up for BM patients. Additionally, the facility played a key role in conducting longitudinal brain volumetric assessments in glioma survivors, where significant reductions in cortical white matter and hippocampal volumes were observed after RT. These examples underscore the facility’s capacity to support a diverse range of multidisciplinary research projects, offering tailored solutions across various imaging modalities (CT, MR, and PET) that significantly improve both the accuracy and efficiency of image analysis while addressing the specific needs of researchers.

A review of the literature on AI-based medical imaging core facilities reveals several key lessons that align with our experiences. For example, studies conducted at Martinos Center for Biomedical Imaging, the Stanford Center for Artificial Intelligence in Medicine & Imaging, NYU Langone Health’s Imaging, Washington University Center for Clinical Imaging Research (CCIR), and Vanderbilt University’s Institute of Imaging Science (VUIIS) highlight the critical role of integrating AI-driven advanced imaging technologies with robust data processing capabilities to enhance research quality and streamline workflows [41,42,43]. While it can be challenging to pinpoint the limitations of different medical imaging core facilities, several key factors essential for their successful operation emerge from these institutions.

First, these institutions emphasize the importance of integrating AI into advanced imaging techniques. For instance, the Martinos Center and NYU Langone Health have developed AI-driven tools to enhance MRI technology and multimodal imaging synthesis, significantly improving the speed and accuracy of image reconstruction and diagnosis. Second, AI has proven invaluable in the clinical translation of disease diagnosis, with tools such as deepMRAC and AI-based imaging biomarkers aiding in conditions like multiple sclerosis, Alzheimer’s disease, and knee osteoarthritis, thereby driving improved treatment outcomes [44,45,46]. Third, data curation and public access to high-quality datasets have been essential for developing AI-based predictive models. The Stanford Center and NYU Langone Health maintain public repositories that support research across diverse conditions such as brain tumors, chest pathologies, and neurological diseases, addressing the challenge of limited datasets by providing curated, openly available clinical imaging data [47,48]. Finally, these facilities stress the importance of overcoming challenges related to AI implementation. Institutions like Washington University and Vanderbilt University emphasize the need for efficient archiving of large datasets, addressing racial subgroup variability, and integrating AI with existing clinical infrastructure such as PACS systems. By streamlining data management and ensuring AI frameworks are accessible, these facilities continue to advance the role of AI in improving patient outcomes across diverse populations [29,49].

Our experience provides valuable insights and perspectives for other institutions looking to establish or refine their image processing and analysis laboratories. Like many of the facilities mentioned earlier, we have successfully integrated AI-driven imaging technologies to enhance workflow efficiency and research quality. This alignment with institutions such as the Martinos Center and NYU Langone Health demonstrates our shared commitment to advancing clinical translation and improving diagnostic accuracy through AI. Moreover, all our projects focus on CT, PET, and MR imaging, where data heterogeneity is well characterized and accounted for during training by collecting a diverse set of patient anatomical profiles and machine/acquisition parameters. AMIPA is designed for scalability; capable of handling large-scale datasets through high-performance computing; and is modality-agnostic, integrating smoothly with clinical workflows such as PACS, MIM, and ARIA. For routine processing, we rely on our dedicated servers, which comfortably handle concurrent tasks. However, for larger-scale or computationally intensive projects—typically those that would exceed a week of processing on our production server—we leverage our HPC computing cluster. This flexible approach allows us to efficiently manage a wide range of workloads, from everyday tasks to large-scale analyses. In contrast with commercial AI tools that offer black-box solutions, AMIPA remains customizable and transparent, enabling modifications based on specific research needs. However, there are still gaps in our facility that need to be addressed. For instance, while we benefit from robust data processing capabilities, we have yet to fully implement the multi-modal image reconstruction models, large-scale data curation, and open access repositories, priorities for facilities like Stanford and NYU Langone Health. All use cases demonstrated in this technical note represent internal, institutional case studies, ensuring data privacy by training models locally. Looking ahead, collaborations involving inter-institutional data may introduce new challenges. However, approaches such as federated learning could be implemented to maintain data privacy [50]. Moreover, the limited availability of medical imaging experts, often occupied with patient care, can delay imaging reviews and impact project timelines. We also recognize the steep learning curve associated with adopting AI tools and the resource-intensive nature of setting up and maintaining an advanced facility. Our experiences, combined with documented findings from other institutions, underscore the need for continuous investment in AI infrastructure and training to bridge these gaps. By comparing our efforts with the successes and challenges faced by other leading facilities, we aim to contribute to a broader understanding of best practices while identifying areas where further refinement and innovation are required to keep pace with the evolving landscape of AI-based medical imaging.

Developing a medical image processing and analysis core facility has the potential to significantly enhance research capabilities and improve clinical workflows, particularly in fields reliant on advanced imaging techniques. However, our experience highlights the substantial resources and ongoing investment required to establish and maintain such a facility, especially in areas like data curation and staff training. By addressing these challenges, we aim to provide a practical framework for other institutions undertaking similar efforts. While the benefits of AI-driven imaging technologies are evident in improved efficiency and diagnostic accuracy, integrating these tools into clinical and research environments is resource-intensive and requires careful, sustained effort. The development of our facility, combined with insights from other institutions, emphasizes the importance of incremental innovation and continuous refinement to keep pace with evolving imaging technologies and their clinical applications.

## Figures and Tables

**Table 1:** Workstations and servers at AMIPA with supercomputing facility at UAB.

Description	Processor	Number of CPU Cores	RAM	GPU(s)	GPU VRAM	Disk Storage	Type
Lambda Vector	Intel^®^ Core™ i9-10900X CPU @ 3.70 GHz × 20	10	128 GB	NVIDIA GeForce RTX 3090	24 GB	1 TB	Development
HPE DL345 Gen10+ 8SFF CTO Svr	AMD EPYC 7443P	24	128 GB	2 x NVIDIA A10	24 GB	1.6 TB	Staging/Production

## Supplementary Materials

The following supporting information can be downloaded at: https://www.mdpi.com/article/10.3390/diagnostics15070803/s1, File S1. Example Software Stack for workflow automation.
