# Supplementary material for: Demonstrating an Academic Core Facility for Automated Medical Image Processing and Analysis: Workflow Design and Practical Applications
Source: Diagnostics (Basel). 2025 Mar 21;15(7):803. doi: 10.3390/diagnostics15070803 (PMC11988328; doi:10.3390/diagnostics15070803)
Supplement: Supplementary file 1 [file diagnostics-15-00803-s001.zip › diagnostics-3484156-Supplementary File S1.pdf]

Supplemental Materials

S1. Example Software Stack for workflow automation.

1. File Management:

   The workflow incorporates solutions that support efficient file storage, version control, and secure data transfer. Examples of such tools include Git-based version control systems (e.g., Git) and secure file transfer protocols (e.g., SFTP). In addition, a lightweight, adaptable file service (e.g., S3-compatible storage) can be configured to handle large datasets while minimizing overhead.

2. Database Management:

   For persistent data storage, the workflow relies on well-established relational database systems (e.g., SQL) and, where necessary, distributed data management frameworks. These tools balance performance and reliability, ensuring that query handling, data integrity, and concurrency are maintained at scale.

3. Scheduling and Orchestration:

   Automated scheduling is facilitated by tools that allow for modularizing tasks, handling dependencies, and supporting distributed execution. Common examples include workflow engines (e.g., Luigi or Redis) that provide fault-tolerance, simplify integration with diverse data processing libraries, and offer advanced logging.

4. Deployment Stack:

   Deployment is performed through container-based approaches (e.g., Docker) and can include standardized orchestration services (e.g., Kubernetes). This arrangement supports seamless updates and integrations, ensuring modular changes do not disrupt the overarching workflow. Additionally, resource provisioning within this framework is dynamically scaled to accommodate fluctuating workloads.

5. Other Relevant Components:

   Additional elements include authentication and access control layers—often employing standard security solutions (e.g., LDAP or SSO frameworks)—to preserve data privacy and user management. Furthermore, auxiliary libraries for monitoring and performance analytics (e.g., time-series log analysis) help optimize workflows without disclosing proprietary metrics or processes.
